# Non-Invasive Detection of Protein Content in Several Types of Plant Feed Materials Using a Hybrid Near Infrared Spectroscopy Model

**DOI:** 10.1371/journal.pone.0163145

**Published:** 2016-09-26

**Authors:** Xia Fan, Shichuan Tang, Guozhen Li, Xingfan Zhou

**Affiliations:** 1 Institute of Quality Standard and Testing Technology for Agro-products of CAAS, Beijing, 100081, China; 2 Beijing Key Laboratory of Occupational Health and Safety, Beijing Municipal Institute of Labor Protections, Beijing, 100054, China; 3 College of Engineering, China Agricultural University, Beijing, 100083, China; Agricultural University of Athens, GREECE

## Abstract

Near-infrared spectroscopy combined with chemometrics was applied to construct a hybrid model for the non-invasive detection of protein content in different types of plant feed materials. In total, 829 samples of plant feed materials, which included corn distillers’ dried grains with solubles (DDGS), corn germ meal, corn gluten meal, distillers’ dried grains (DDG) and rapeseed meal, were collected from markets in China. Based on the different preprocessed spectral data, specific models for each type of plant feed material and a hybrid model for all the materials were built. Performances of specific model and hybrid model constructed with full spectrum (full spectrum model) and selected wavenumbers with VIP (variable importance in the projection) scores value bigger than 1.00 (VIP scores model) were also compared. The best spectral preprocessing method for this study was found to be the standard normal variate transformation combined with the first derivative. For both full spectrum and VIP scores model, the prediction performance of the hybrid model was slightly worse than those of the specific models but was nevertheless satisfactory. Moreover, the VIP scores model obtained generally better performances than corresponding full spectrum model. Wavenumbers around 4500 cm^-1^, 4664 cm^-1^ and 4836 cm^-1^ were found to be the key wavenumbers in modeling protein content in these plant feed materials. The values for the root mean square error of prediction (*RMSEP*) and the relative prediction deviation (*RPD*) obtained with the VIP scores hybrid model were 1.05% and 2.53 for corn DDGS, 0.98% and 4.17 for corn germ meal, 0.75% and 6.99 for corn gluten meal, 1.54% and 4.59 for DDG, and 0.90% and 3.33 for rapeseed meal, respectively. The results of this study demonstrate that the protein content in several types of plant feed materials can be determined using a hybrid near-infrared spectroscopy model. And VIP scores method can be used to improve the general predictability of hybrid model.

## Introduction

Due to the shortage of protein-based feed materials, plant feed materials, including corn distillers’ dried grain with solubles (DDGS), corn germ meal, corn gluten meal, distillers’ dried grains (DDG) and rapeseed meal, are popularly used in China. Most of these plant feed materials are byproducts, and their nutrient profiles, particularly the protein content, can vary significantly with different raw materials, production years, production routes and production factors, etc. [1]. The protein content in feed materials is essential for livestock diet formulation and is a major determinant of the feed price. However, determining the protein content using the wet-chemistry laboratory method is time-consuming and costly. In contrast, near-infrared reflectance (NIR) spectroscopy is a rapid, non-invasive, reliable and environmentally friendly detection technology and has been successfully used to determine the protein content in many feed materials [2–4].

Normally, for a single type of sample, a specific NIR spectroscopy calibration model (specific model) will be built, while if several types of samples are obtained, several specific models are required. However, the optimization of the modeling parameters, such as the calibration set, spectral preprocessing, regression algorithm and latent variable selection, for a large number of specific models is very exhausting. Moreover, maintenance of several calibration models could be laborious and time-consuming [5]. It would be very convenient and cost-effective if the models for different types of samples could be combined into a single calibration model (hybrid model); thus, the protein content of different types of samples could be predicted using one hybrid model.

Partial least square (PLS) regression is the mostly used method to develop a quantitative model. VIP (variable importance for the projection) scores method is often used to indicate the importance of spectral variables in PLS modeling [6]. Previously studies showed that using VIP scores indicated important variables (VIP scores value bigger than 1) to develop new model could improve prediction performance [7, 8].

In this study, the potential of constructing a hybrid model to assess the protein content in several types of plant feed materials was investigated. The performances of VIP scores method in optimizing the specific and hybrid model were also evaluated.

## Materials and Methods

### Sampling and chemical analysis

A total of 829 samples of plant feed materials, which included corn DDGS (N = 196), corn germ meal (N = 97), corn gluten meal (N = 198), DDG (N = 73) and rapeseed meal (N = 265), were collected from 23 provinces of China in 2008–2013. All feed materials were directly collected from public market in different provinces and no specific permissions were required for the locations/activities. Each sample was well mixed, ground using a Retsch ZM 100 mill (Retsch GmbH, Haan, Germany) and sieved through a 1.00-mm sieve for further analysis.

The protein content was analyzed according to the standard analytical method for feedstuff (GB/T 6432–94) [9] using a Kjeltec 2300 analyzer (FOSS Tecator AB, Höganäs, Sweden) with two duplicates for each sample.

### NIR spectral data collection

Prior to the NIR spectral data collection, the samples were maintained at room temperature (25°C±1°C) for 24 hours, with the temperature controlled by an air-conditioning system. The spectral data were recorded using a NIRflex N-500 FT-NIR spectrometer (Buchi Analytical Inc., New Castle, DE, USA) in the diffuse reflectance mode at room temperature. Approximately 75 g of each sample was poured into a standard quartz cup (10 cm in diameter and 1 cm high) on a spinner using the Integrating Sphere module of the spectrometer. The spectrum of each sample was recorded in triplicate by accumulating 32 scans at a resolution of 8 cm^−1^ between 10,000 cm^−1^ and 4000 cm^−1^. The replicate spectra of each sample were averaged before calibration. Finally, for each sample, one averaged spectrum with 1501 variables was obtained.

### Sample set selection

For each type of feed material, all the spectral data were sorted in ascending order according to the protein content of the samples. The first, third and fourth samples of every four samples were selected as the calibration set samples, whereas the remaining samples were ascribed to the external validation set [2]. All the samples of the calibration set and external validation set that were used in the different specific models were used as the samples for the calibration set and external validation set, respectively, for the hybrid model. The spectral data, protein content and sample set information of all the samples were summarized in S1 Data.

### Modeling

To remove or minimize the noise and enhance the spectral features, the standard normal variate (SNV) and SNV with the 1^st^ or 2^nd^ derivative (9-point Savitzky-Golay filter and a second-order polynomial fit) (SNVD1 or SNVD2) preprocessing methods were applied. And all the spectral data were autoscaled before final modeling.

To measure certain spectral variables or wavenumbers that are important for partial least-squares regression modeling, the VIP scores were used [10], which are defined as follows:

$${\text{VIP}}_{\text{j}}=\sqrt{N\frac{\sum_{K=1}^{F}(b_{k}^{2}t_{k}^{T}t_{}){\left(w_{jk}/W_{k}\right)}^{2}}{\sum_{k=1}^{F}(b_{k}^{2}t_{k}^{t}{t)}_{}}},$$ (1)

where *F* is the number of latent vectors (LVs) for the model, ***t***_k_ is the vector of sample scores along the *k*^th^ PLS inner relationship, *N* is the number of variables, and *w*_jk_ and ***W***_k_ are the weight of the *j*^th^ variable and the weight vector for the *k*^th^ LV, respectively. For all the spectral variables, the average of the squared VIP scores is equal to 1. The variables with VIP scores greater than 1 are generally accepted as significant variables for modeling.

The spectral data were preprocessed and modeled on the MATLAB 2012b platform (The MathWorks, Inc., Natick, MA, USA) with the PLS toolbox (version 6.71, Eigenvector Research, Inc., USA).

### Model evaluation

The coefficient of determination for calibration (*R*^*2*^_c_), root mean square error of calibration (*RMSEC*), coefficient of determination for cross validation (*R*^*2*^_*cv*_), root mean square error of cross validation (*RMSECV*), coefficient of determination for validation (*r*^*2*^_v_), root mean square error of prediction (*RMSEP*) and the relative prediction deviation (*RPD*, which is defined as *SD*/*RMSEP*, where *SD* denotes the standard deviation) were calculated to evaluate the NIR model performance. Commonly, a higher *RPD* value corresponds to a greater predictability of the calibration model. Specifically, an *RPD* value between 2.0 and 2.5 indicates that an approximate quantitative prediction is possible, while an *RPD* value of 2.5–3.0 reveals that the calibration model has good prediction accuracy, and an *RPD* value above 3.0 suggests that the calibration model has excellent prediction accuracy [11, 12].

## Results and Discussion

### Protein content

The protein content of the samples as determined by wet-chemistry laboratory analysis had a standard error below 0.36% in the laboratory measurements. Table 1 summarizes the protein content of different plant feed materials in the different sample sets.

**Table 1:** Summary of protein content in calibration set and validation set of different plant feed materials.

Materials	Protein content % of materials (as received)										
	Calibration set					Validation set					*SEL*
	*No*.	Mean	Range	*SD*	*CV*	*No*.	Mean	Range	*SD*	*CV*	
Corn DDGS	147	28.09	20.46–33.32	2.57	9.15	49	28.11	20.85–33.22	2.66	9.46	0.36
Corn germ meal	73	19.91	10.64–30.35	4.02	20.19	24	19.83	10.74–29.96	4.09	20.63	0.15
Corn gluten meal	149	60.87	45.16–70.42	4.67	7.67	49	60.74	45.85–68.29	4.74	7.80	0.20
DDG	55	24.45	12.01–38.69	7.17	29.33	18	24.01	12.24–35.28	7.07	29.45	0.17
Rapeseed meal	199	38.35	29.77–44.34	2.99	7.79	66	38.34	30.83–43.59	2.99	7.80	0.35

*No*.: number of samples; *CV*: Coefficient of variation [(SD/mean) ×100]. *SD*: standard deviation; *SEL*: standard error in laboratory determinations.

Previous studies reported that the protein content of corn DDGS, corn germ meal, DDG and rapeseed meal were in the ranges of 20%-33% [2], 21%-25% [13], 12%-38% [14] and 29%-40% [15], respectively. The samples collected in this study covered the protein content ranges for all four types of samples, which indicates good sampling representativeness. Regarding to corn gluten meal, the mean protein content of collected samples is 61%, which is similar to that reported in reference (61%) [16].

### Spectra

**Fig 1:**
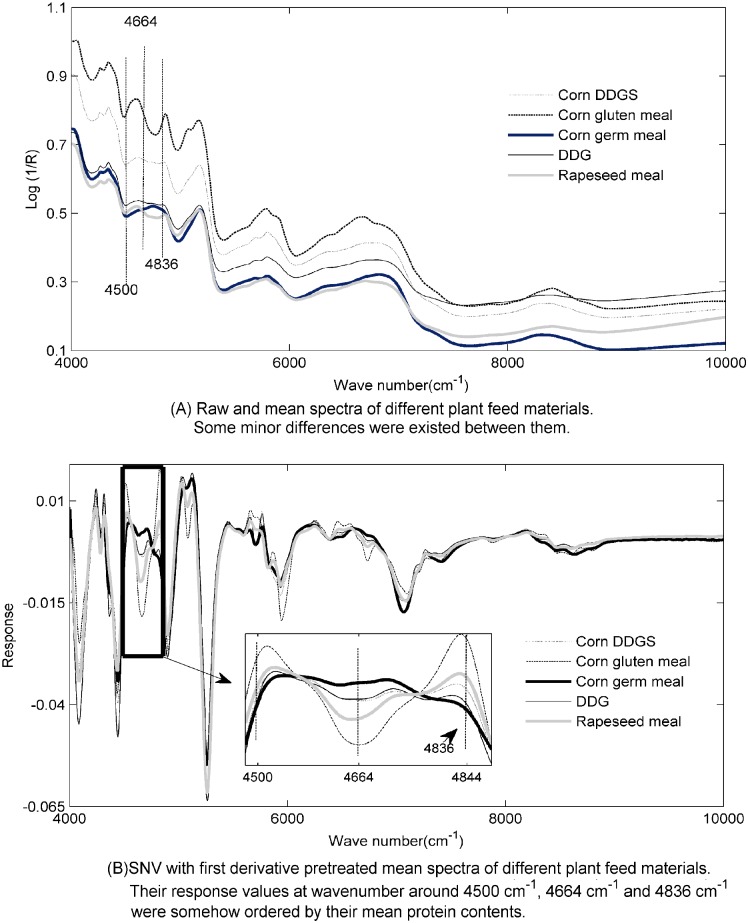
Raw and pretreated spectra for different types of plant feed materials. (A) Raw and mean spectra of different plant feed materials. Some minor differences were existed between them. (B) SNV with first derivative pretreated mean spectra of different plant feed materials. Their response values at wavenumber around 4500 cm^-1^, 4664 cm^-1^ and 4836 cm^-1^ were somehow ordered by their mean protein contents.

Raw and pretreated spectra for different types of plant feed materials were presented in Fig 1A and 1B, respectively. The raw spectra of each types of plant feed materials were generally similar but some minor differences were existed. For example, spectra of corn DDGS, corn germ meal and DDG were nearly flat from 4664 cm^-1^ to 4836 cm^-1^. While that of corn gluten meal and rapeseed meal were not flat, a valley can be visually observed at those wavenumbers. Moreover, some differences can be directly found with regard to their SNVD1 pretreated mean spectra. It’s interesting to see that the response values at wavenumbers around 4500 cm^-1^, 4664 cm^-1^ and 4836 cm^-1^ were somehow ordered by the mean protein content of different types of plant feed materials. Moreover, according to the reference [17], 4500 cm^-1^, 4664 cm^-1^ and 4836 cm^-1^ are closely associated with vibrations of proteins. These results indicated that aforementioned wavenumbers may be play important roles in modeling protein content in those samples.

### Full spectrum specific NIR models

Specific models were constructed with full NIR spectral data that were preprocessed using SNV, SNVD1 and SNVD2. The statistical evaluation of the performance of the optimized specific NIR models is summarized in Table 2. The results indicate that the specific NIR model based on the SNVD1 preprocessed data was the most accurate model among those evaluated. This result suggests that SNVD1 preprocessing may be the most suitable preprocessing method to remove the noise in the spectral data of plant feed samples. Except for corn DDGS (*RPD* = 2.96), all of the specific models yielded excellent prediction results (*RPD*>3).

**Table 2:** Results of optimal NIR quantification models for different kinds of plant feed materials.

Model	Materials	Variable number	LV (variance)	Calibration set				Validation set		
				*R*^2^_c_	*RMSEC*	*R*^*2*^_*cv*_	*RMSECV*	*r*^*2*^_*v*_	*RMSEP*	*RPD*
Full spectrum specific model	Corn DDGS	1501	6(82.52%)	0.91	0.76	0.88	0.89	0.89	0.90	2.96
	Corn germ meal	1501	5(84.22%)	0.97	0.67	0.96	0.83	0.98	0.78	5.24
	Corn gluten meal	1501	3(67.88%)	0.98	0.65	0.98	0.70	0.98	0.64	7.40
	DDG	1501	4(82.04%)	0.97	1.29	0.95	1.63	0.93	1.86	3.80
	Rapeseed	1501	4(80.66%)	0.93	0.78	0.92	0.83	0.93	0.78	3.83
Full spectrum hybrid model	Corn DDGS			0.87	0.99	0.86	1.02	0.86	1.01	2.63
	Corn germ meal			0.91	1.24	0.90	1.28	0.90	1.39	2.94
	Corn gluten meal			0.94	1.14	0.94	1.17	0.94	1.13	4.64
	DDG			0.92	1.37	0.91	1.48	0.92	1.53	4.62
	Rapeseed meal			0.91	0.90	0.90	0.92	0.92	0.86	3.48
	All materials	1501	6(89.15%)	0.99	1.08	0.99	1.13	0.99	1.10	14.77
VIP scores specific model	Corn DDGS	342	7(91.64%)	0.92	0.74	0.89	0.86	0.91	0.79	3.37
	Corn germ meal	651	5(94.22%)	0.97	0.64	0.96	0.84	0.97	0.75	5.45
	Corn gluten meal	553	3(82.64%)	0.98	0.70	0.97	0.75	0.98	0.63	7.52
	DDG	326	4(80.89%)	0.96	1.41	0.94	1.76	0.95	1.67	4.23
	Rapeseed	565	4(85.05%)	0.93	0.78	0.92	0.82	0.93	0.77	3.88
VIP scores hybrid model	Corn DDGS			0.87	0.96	0.85	1.03	0.86	1.05	2.53
	Corn germ meal			0.94	1.01	0.93	1.08	0.95	0.98	4.17
	Corn gluten meal			0.96	0.87	0.96	0.92	0.98	0.75	6.99
	DDG			0.96	1.36	0.95	1.61	0.96	1.54	4.59
	Rapeseed meal			0.90	0.93	0.90	0.95	0.91	0.90	3.33
	All materials	544	7(94.71%)	0.99	0.98	0.99	1.05	0.99	0.99	16.41

For all type of models, the optimal spectral pretreatment method is standard normal variate with 1^st^ derivative; *R*^*2*^_c_: the coefficient of determination for the calibration; *RMSEC*: Root mean square error of calibration; *R*^2^_cv_: the coefficient of determination for the cross validation; *RMSECV*: Root mean square error of cross validation; *r*^*2*^_v_: the coefficient of determination for the validation; *RMSEP*: Root mean square error of prediction; *RPD*: the residual predictive deviation (*RPD* = *SD*/*RMSEP*).

**Fig 2:**
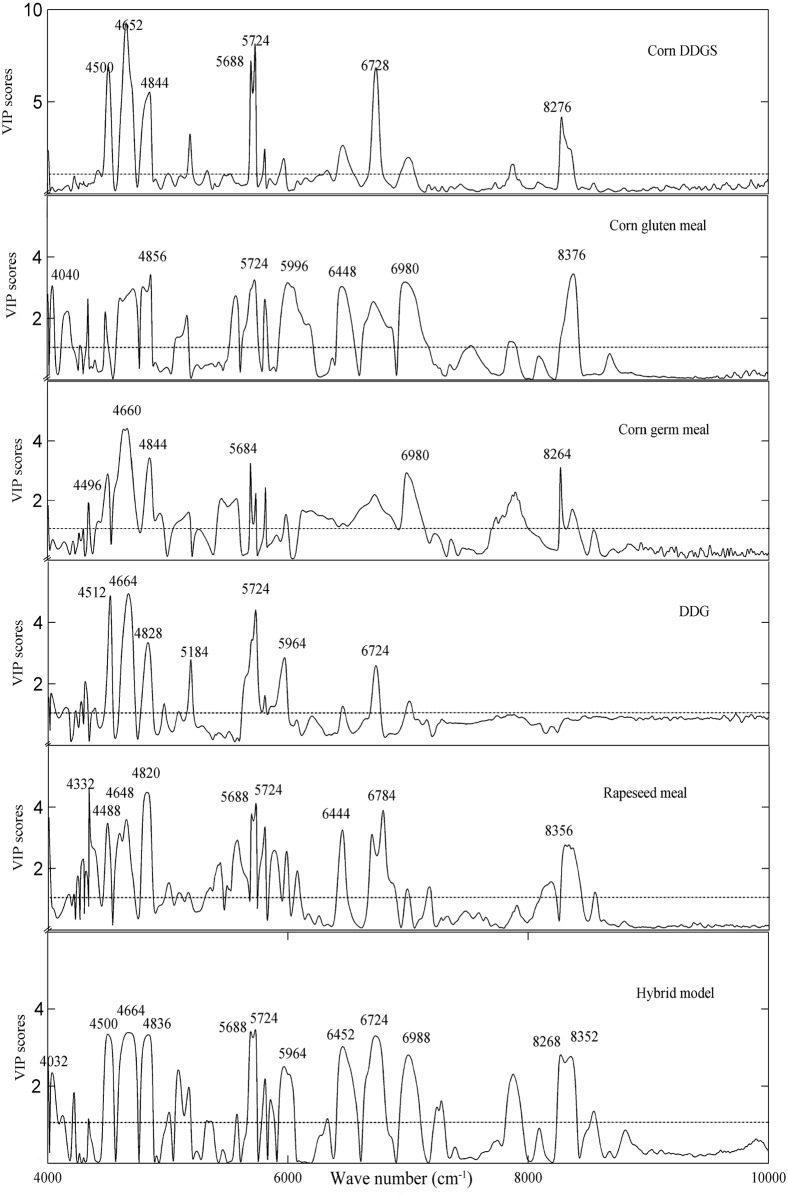
VIP scores curves for full spectrum specific and hybrid models.

Fig 2 displays the VIP scores plots of the specific models for different plant feed materials. Clearly, the wavenumbers of approximately 4500 cm^-1^, 4660 cm^-1^, 4836 cm^-1^, 5684 cm^-1^, 5724 cm^-1^ and 6728 cm^-1^ contribute the most to modeling the protein content in these plant feed ingredients. These wavenumbers are closely related to the chemical structure of proteins; specifically, 4500 cm^-1^ and 4660 cm^-1^ are associated with the combination of the N-H, C-N and C = O vibrations of the amide group; 4836 cm^-1^ is associated with the N-H vibration of proteins; 5684 cm^-1^ and 5724 cm^-1^ are associated with the C-H vibration of lipids, respectively, and 6728 cm^-1^ is associated with the N-H vibration of aromatic amines [17].

However, different plant feed materials had distinctive VIP scores peaks, even for materials with the same origin. For example, the most important wavenumbers that contributed primarily to modeling the protein content in corn DDGS were 4500 cm^-1^, 4652 cm^-1^, 4844 cm^-1^, 5688 cm^-1^, 5724 cm^-1^, 6728 cm^-1^ and 8276 cm^-1^, whereas those for corn gluten meal were 4040 cm^-1^, 4856 cm^-1^, 5724 cm^-1^, 5996 cm^-1^, 6448 cm^-1^, 6980 cm^-1^ and 8376 cm^-1^. According to the applicable reference [17], 4040 cm^-1^ can be associated with the C-N-C vibration of proteins or C-H vibration of cellulose and starch; 4500 cm^-1^, 4844 cm^-1^ and 4856 cm^-1^ are attributed to the N-H vibration of proteins; 5996 cm^-1^ are associated with the C-H vibration of ketones; 5688 cm^-1^ and 8376 cm^-1^ are attributed to the C-H vibration of the lipids, 6448 cm^-1^ can be assigned to O-H vibration of water or N-H vibration of proteins; and 4652 cm^-1^, 6728 cm^-1^ and 6980 cm^-1^ are associated with C-H or N-H vibration of aromatic amides. These results indicate that corn DDGS and corn gluten meal significantly differ in protein content, more specifically, in the aliphatic and aromatic amino acid contents. Such a large difference in the protein content between corn DDGS (28.09%) and corn gluten meal (60.87%) is clearly illustrated in Table 1. The data from the Chinese Feed Database confirmed that the average contents of leucine (3.21% vs. 10.50%, aliphatic amino acid), phenylalanine (1.40% vs. 3.94%, aromatic amino acid) and tyrosine (1.09% vs. 3.19%, aromatic amino acid) in corn DDGS and corn gluten meal (27.50% vs. 63.50%, protein) are also notably different [18]. These results imply that distinctive VIP scores peaks of different plant feed materials can be used to express their chemical composition characteristics.

### Full spectrum hybrid NIR models

Similarly, hybrid models were also constructed using the full NIR spectral data and different preprocessing methods, and the model that was preprocessed with SNVD1 yielded the best results (see Table 2). The *R*^*2*^_c_, *r*^*2*^_v_, *RMSEC*, *RMSEP* and *RPD* for the optimal hybrid model were 0.99, 0.99, 1.08%, 1.17% and 14.77, respectively.

**Fig 3:**
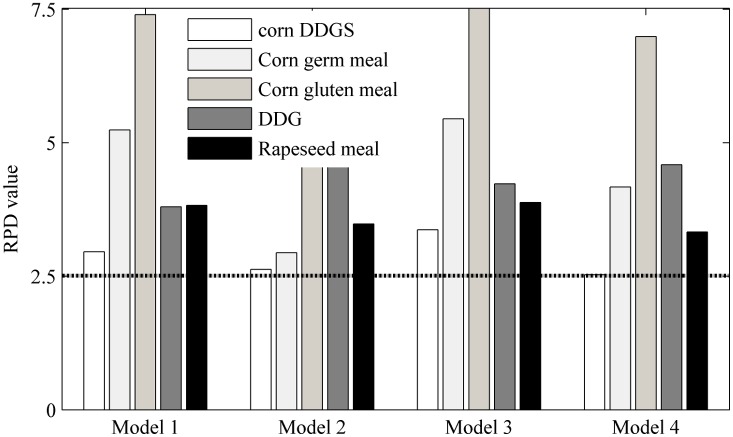
The RPD value for different kinds of plant feed materials in different NIR models. Model 1 to Model 4 stand for full spectrum specific models, full spectrum hybrid model, VIP scores specific models and VIP scores hybrid model, respectively.

The *RPD* values that were obtained using different NIR models for each type of plant feed material are presented in Fig 3. The general performance of the hybrid model for each material was slightly worse than those of the specific models. The *RPD* values for corn DDGS, corn germ meal, corn gluten meal and rapeseed meal decreased by 11.15%, 43.89%, 37.57% and 9.14%, respectively. Notably, the *RPD* value of DDG increased by 21.58%.

For pure material such as corn gluten meal, with sufficient calibration set samples, the protein related spectral information could be successfully extracted using the corresponding specific model with excellent prediction accuracy (as shown in Table 1, *RPD* = 7.40). However, in the hybrid model, except for the information from corn gluten meal, the spectral information from other types of plant feed ingredients were also involved. This information could not be discarded because these data played important roles in modeling the protein content of other types of materials. However, these data provide redundant information for modeling the protein content in corn gluten meal. As such, a reduction in the prediction accuracy for corn gluten meal was inevitable. Such was also the case with corn DDGS, corn germ meal and rapeseed meal. Fig 2 clearly indicates that VIP scores plot of the hybrid model was different from that of each specific model. Because the VIP scores closely define the protein composition characteristics of each type of plant feed material, the inconsistency of the VIP scores plots between a hybrid model and the specific models from which it is derived also explains to some extent why the hybrid model did not perform as well as the specific models.

In contrast, DDG is a byproduct from the brewer’s fermentation industry, which contains ingredients such as corn, wheat, and sorghum [14]. The complexity of ingredients and the relatively limited calibration samples (N = 55) may preclude extracting protein-related spectral information from the specific model. The specific model may fail to achieve perfect prediction accuracy. However, the information from other samples in addition to DDG, particularly the information from samples of corn origin, such as the corn DDGS, corn germ meal and corn gluten meal, are beneficial for modeling DDG. Thus, it is reasonable that the prediction accuracy of the DDG content in the hybrid model was increased.

### VIP scores specific and hybrid model

By using those important variables (VIP scores value >1.0) indicated by VIP scores method, corresponding new specific models (VIP scores specific model) and hybrid model (VIP scores hybrid model) were developed. And related results were summarized in Table 2. Results showed that all five VIP scores specific models developed with less spectral variables but obtained better prediction results than their corresponding full spectrum models, respectively. In regard to VIP scores hybrid model, its *R*^*2*^_c_, *r*^*2*^_v_, *RMSEC*, *RMSEP* and *RPD* were 0.99, 0.99, 1.05%, 0.99% and 16.41, respectively. The prediction performance is general better than the full spectrum hybrid model. These results showed that VIP scores method could improve prediction performance both for specific models and hybrid model. Moreover, similar to those full spectrum models, the performance of VIP scores hybrid model for each material was slightly worse than those of the VIP scores specific models, except for DDG.

**Fig 4:**
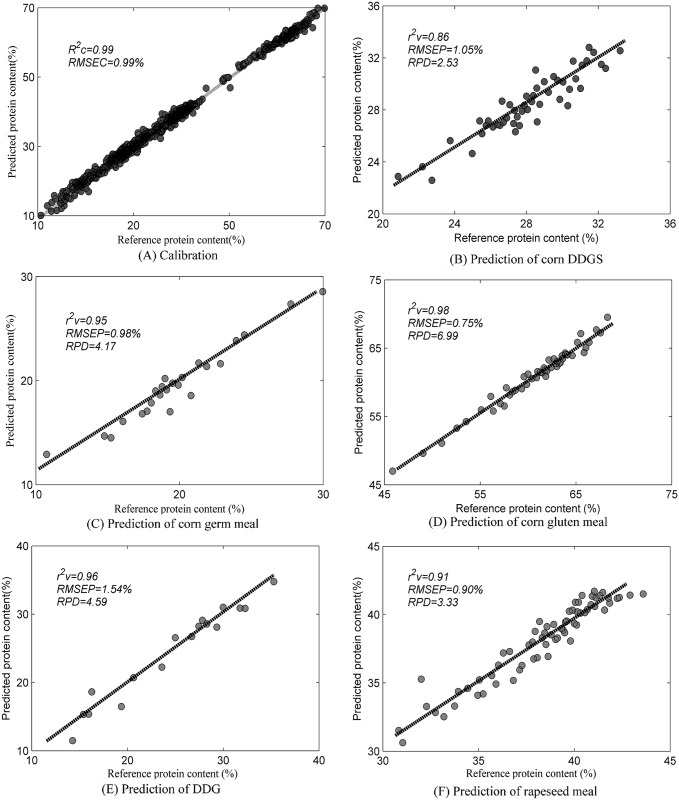
Calibration and validation results of VIP scores hybrid model. This hybrid model was constructed by 544 variables with VIP scores value bigger than 1.

The values for *RMSEP* and the *RPD* obtained with the VIP scores hybrid model were 1.05% and 2.53 for corn DDGS, 0.98% and 4.17 for corn germ meal, 0.75% and 6.99 for corn gluten meal, 1.54% and 4.59 for DDG, and 0.90% and 3.33 for rapeseed meal, respectively. Fig 4 is the scatter plot of the protein values that were determined using the VIP scores hybrid NIR model fitted to the reference protein content of the calibration set and validation set samples. There is very good agreement between the hybrid NIR fit and the reference data.

The VIP scores hybrid model performance with corn DDGS (*RPD* = 2.53) and DDG (*RPD* = 4.59) were slightly worse than that reported in previous studies (*RPD* = 3.42 [2] and *RPD* = 4.98 [14]). The VIP scores hybrid model performed better for rapeseed meal (*RMSEP* = 0.90%) than that reported by Daszykowski et al. (*RMSEP* = 1.12%) [15]. In general, the hybrid performance for each type of plant feed ingredient was satisfactory (*RPD*>2.5). These results indicate that a hybrid NIR model can be constructed to predict the contents of different types of materials. Although these results are encouraging, further development is required to validate the effectiveness and robustness of this type of hybrid model using more samples from the existing and new materials. Both the differences detected from SNVD1 pretreated spectra and the VIP scores peaks detected from VIP scores curves of different full spectrum models implied that wavenumbers around 4500 cm^-1^, 4664 cm^-1^ and 4836 cm^-1^ are closely related to protein content of these plant feed materials. And these three wavenumbers are found to be specifically associated with vibrations of proteins. As such, a hybrid model with these three wavenumbers was built and the results were shown in S1 Table. This model gave out a rough estimate of protein content in different kind of plant feed materials, with the range of *RMSEP* and *RPD* values were 1.64%-4.18% and 1.28–2.03, respectively. Though the prediction accuracy was not satisfactory, it’s still confirmed that 4500 cm^-1^, 4664 cm^-1^ and 4836 cm^-1^ are key wavenumbers in modeling protein content of these plant feed materials.

## Conclusions

This paper evaluates the potential of near-infrared spectroscopy combined with chemometrics in constructing a hybrid model for the non-invasive detection of protein content in different types of plant feed ingredients. The results reveal that it is feasible to detect the protein content in corn DDGS, corn germ meal, corn gluten meal, DDG and rapeseed meal using a hybrid near-infrared spectroscopy model. VIP scores method is a powerful means which can detect important variables for modeling and improve prediction performances for both specific models and hybrid model. Wavenumbers around 4500 cm^-1^, 4664 cm^-1^ and 4836 cm^-1^ are found to be key wavenumbers in modeling protein content of these plant feed materials.

## Supporting Information

S1 Data The spectral data, protein content and sample set information of all the samples. All the data were stored as matlab files. Each matlab file was named by the type of plant feed materials. In the matlab data matrix, each row stands for a sample. The 1^st^ column indicates the sample set of each sample, 0 indicates it belongs to the calibration set, whereas 1 respresents it belongs to the external validation set. The 2^nd^ column indicates the protein content of each sample. The 3^rd^ column to the 1503^th^ column indicate the spectral data (4000 cm^-1^ to 10000 cm^-1^).
(MAT)

S1 Table Results of hybrid models constructed with three most important variables. This model gave out a rough estimate of protein content in different kind of plant feed materials which confirmed that wavenumbers 4500 cm^-1^, 4664cm^-1^ and 4836 cm^-1^ are key wavenumbers in modeling protein content of these plant feed materials. *R*^*2*^_c_: the coefficient of determination for the calibration; *RMSEC*: Root mean square error of calibration; *r*^*2*^_v_: the coefficient of determination for the validation; *RMSEP*: Root mean square error of prediction; *RPD*: the residual predictive deviation (*RPD* = *SD*/*RMSEP*).
(DOC)
