# Supplementary material for: Non-Invasive Detection of Protein Content in Several Types of Plant Feed Materials Using a Hybrid Near Infrared Spectroscopy Model
Source: PLoS One. 2016 Sep 26;11(9):e0163145. doi: 10.1371/journal.pone.0163145 (PMC5036844; doi:10.1371/journal.pone.0163145)
Supplement: S1 Table — R2c: the coefficient of determination for the calibration; RMSEC: Root mean square error of calibration; r2v: the coefficient of determination for the validation; RMSEP: Root mean square error of prediction; RPD: the residual predictive deviation (RPD = SD/RMSEP). (DOC) [file pone.0163145.s002.doc]

**S1 Table Results of hybrid models constructed with three most important variables. This model gave out a rough estimate of protein content in different kind of plant feed materials which confirmed that wavenumbers 4500 cm^-1^, 4664cm^-1^ and 4836 cm^-1^ are key wavenumbers in modeling protein content of these plant feed materials.**

| Materials | Calibration set | | Validation set | | |
| --- | --- | --- | --- | --- | --- |
|  | *R*^2^_c_ | *RMSEC* | *r^2^_v_* | *RMSEP* | *RPD* |
| Corn DDGS | 0.71 | 2.16 | 0.67 | 2.07 | 1.28 |
| Corn germ meal | 0.77 | 2.40 | 0.92 | 2.01 | 2.03 |
| Corn gluten meal | 0.68 | 2.85 | 0.69 | 2.74 | 1.91 |
| DDG | 0.85 | 3.73 | 0.83 | 4.18 | 1.69 |
| Rapeseed meal | 0.70 | 1.64 | 0.74 | 1.64 | 1.83 |
| All materials | 0.97 | 2.41 | 0.97 | 2.38 | 6.83 |

*R^2^*_c_: the coefficient of determination for the calibration;

*RMSEC*: Root mean square error of calibration;

*r^2^*_v_: the coefficient of determination for the validation;

*RMSEP*: Root mean square error of prediction;

*RPD*: the residual predictive deviation (*RPD*=*SD*/*RMSEP*);
